# Assessment of Hyperosmolar Blood–Brain Barrier Opening in Glioblastoma via Histology with Evans Blue and DCE-MRI

**DOI:** 10.3390/biomedicines11071957

**Published:** 2023-07-11

**Authors:** Jérôme Conq, Nicolas Joudiou, Bernard Ucakar, Kevin Vanvarenberg, Véronique Préat, Bernard Gallez

**Affiliations:** 1 UCLouvain, Louvain Drug Research Institute (LDRI), Biomedical Magnetic Resonance Research Group, 1200 Brussels, Belgium; jerome.conq@uclouvain.be; 2 UCLouvain, Louvain Drug Research Institute (LDRI), Advanced Drug Delivery and Biomaterials Research Group, 1200 Brussels, Belgium; bernard.ucakar@uclouvain.be (B.U.); kevin.vanvarenberg@uclouvain.be (K.V.); veronique.preat@uclouvain.be (V.P.); 3 UCLouvain, Louvain Drug Research Institute (LDRI), Nuclear and Electron Spin Technologies (NEST) Platform, 1200 Brussels, Belgium; nicolas.joudiou@uclouvain.be

**Keywords:** blood–brain barrier, glioblastoma, osmotic shock, mannitol, DCE-MRI, Evans blue

## Abstract

Background: While the blood–brain barrier (BBB) is often compromised in glioblastoma (GB), the perfusion and consequent delivery of drugs are highly heterogeneous. Moreover, the accessibility of drugs is largely impaired in the margins of the tumor and for infiltrating cells at the origin of tumor recurrence. In this work, we evaluate the value of methods to assess hemodynamic changes induced by a hyperosmolar shock in the core and the margins of a tumor in a GB model. Methods: Osmotic shock was induced with an intracarotid infusion of a hypertonic solution of mannitol in mice grafted with U87-MG cells. The distribution of fluorescent dye (Evans blue) within the brain was assessed via histology. Dynamic contrast-enhanced (DCE)-MRI with an injection of Gadolinium-DOTA as the contrast agent was also used to evaluate the effect on hemodynamic parameters and the diffusion of the contrast agent outside of the tumor area. Results: The histological study revealed that the fluorescent dye diffused much more largely outside of the tumor area after osmotic shock than in control tumors. However, the study of tumor hemodynamic parameters via DCE-MRI did not reveal any change in the permeability of the BBB, whatever the studied MRI parameter. Conclusions: The use of hypertonic mannitol infusion seems to be a promising method to increase the delivery of compounds in the margins of GB. Nevertheless, the DCE-MRI analysis method using gadolinium-DOTA as a contrast agent seems of limited value for determining the efficacy of opening the BBB in GB after osmotic shock.

## 1. Introduction

Glioblastoma (GB) is the most common and aggressive malignant tumor of the central nervous system in adults, accounting for more than 50% of gliomas. The current standard of care is to remove as much tumor tissue as possible via surgical resection, followed by adjuvant radiotherapy and chemotherapy to eradicate residual infiltrating tumor cells at the origin of recurrences [1]. Despite this aggressive treatment, the prognosis of patients suffering from GB remains poor, with a high recurrence rate, a median survival time lower than 2 years, and a 5 years survival rate lower than 5% [2,3].

This tumor shows a high proliferation rate, intratumoral and intertumoral heterogeneity, highly invasive and infiltrative cell properties in the adjacent brain parenchyma, and resistance to chemotherapy, making GB a very challenging cancer to treat. Many distinctive features of GB (genetic, cytological, or anatomical) hinder the treatment efficacy and must be overcome. One of the fundamental issues for treating GB is linked to the blood–brain barrier (BBB). This physiological barrier controls the entry of compounds into the brain and significantly inhibits the passage of drugs from the bloodstream to the brain, reducing the effectiveness of chemotherapeutic agents’ systemic delivery [4]. While the BBB is often compromised in GB, the perfusion and consequent delivery of drugs are highly heterogeneous. Indeed, tumoral BBB is characterized by an aberrant distribution of pericytes, a loss of astrocytic end-feet, as well as a loss of tight junction proteins between endothelial cells. Unlike the healthy BBB, the leaky tumoral BBB allows the passage of large molecules between endothelial cells, where the paracellular pathway is damaged [5]. However, tumoral BBB permeability is highly heterogeneous and varies not only between GB but also within the same tumor, with tumor regions (mainly in the margins of the tumor) where the BBB is still intact, which limit the access of drugs to the infiltrating tumor cells at the origin of the tumor recurrence [6,7]. Therefore, the tumoral BBB remains a major hurdle to overcome.

**Figure 1:**
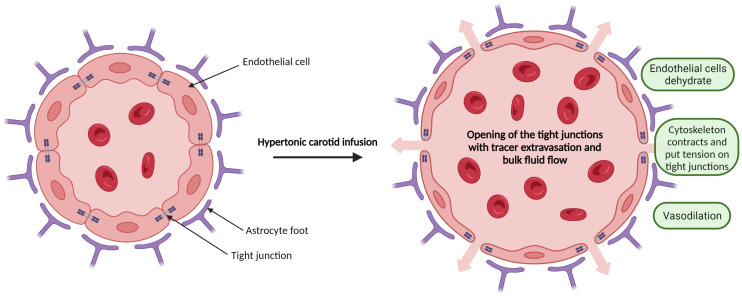
Model for the opening of the inter-endothelial tight junctions via carotid infusion of a hypertonic solution. During infusion, endothelial cells dehydrate, water leaves the brain, leading to vasodilation, and the endothelial cell cytoskeleton contracts. Tension is exerted at tight junctional areas due to each of these factors, leading to a reversible opening of the junctions [9].

Many efforts have been made in past decades to develop new approaches in order to overcome it and improve the delivery of drugs to the tumor, especially in its margins. One of the strategies that we will examine deeper in this article is osmotic shock. Previous works have shown that hypertonic solution infusion induces a reversible opening of a healthy BBB [8,9]. The mechanism of action of this method is explained in Figure 1.

In this work, our aim was to evaluate whether this strategy can be used for tumoral BBB opening in GB, especially for the margins of the tumor. To do so, we evaluated whether a hyperosmolar shock may change hemodynamic parameters in the core and the margins of the tumor in a GB model. Two methods have been used to evaluate the efficacy of the treatment on perfusion/permeability parameters: a non-invasive one, based on magnetic resonance imaging (MRI) analysis (T_1_-weighted and T_2_-weighted imaging as well as dynamic contrast-enhanced (DCE)-MRI), and an invasive one, based on histological analysis with a fluorescent vascular leakage marker (Evans blue).

For the MRI analysis, we determined the core of the tumor through the use of T_2_-weighted contrast. In addition, T_1_-weighted images were obtained after the administration of a contrast agent to analyze the diffusion of the contrast agent within the bulk of the tumor and in its margins. Gadolinium-DOTA was used because this hydrophilic contrast agent is unable to cross the intact BBB, offering the possibility to tackle changes in BBB permeability. We defined the tumor margins as the difference between the area of the T_1_-enhancing region after the injection of the contrast agent and the area depicted by the T_2_-weighted contrast delineating the core of the tumor. The hypothesis was that, if there is an increase in the permeability of the BBB, the contrast agent should pass the barrier more easily and diffuse much more largely outside of the tumor area into the brain parenchyma. As a consequence, a larger contrast uptake area should be seen in the T_1_-weighted post-contrast image compared to the T_2_-weighted anatomical image. In other words, an increase in T_1_ in the T_2_ tumor surface ratio would indicate the opening of the BBB. In addition, DCE-MRI analysis gave access to complementary tumor hemodynamic parameters that may be biomarkers of the change in BBB permeability [10].

For the histological analysis, BBB integrity and vascular leakage were assessed by perfusing mice with Evans blue fluorescent dye which binds to plasma albumin with a very high affinity. Albumin cannot cross an intact BBB. However, when compromised, albumin-bound Evans blue can diffuse into the brain parenchyma and be visualized by fluorescence [11].

## 2. Materials and Methods

### 2.1. Orthotopic U-87MG Mouse Model 

All experiments were performed in accordance with the European Directive 2010/63/EU and following the Belgian national regulation guidelines, and were approved by the ethical committee for animal care by the Faculty of Medicine of the Université Catholique de Louvain (2019/UCL/MD/004). Water and food were given ad libitum. Animal body weight was constantly monitored throughout the experiment.

Six-week-old female NMRI nude mice (Janvier, France) were anesthetized via intraperitoneal injection of ketamine/xylazine (100 and 13 mg/kg, respectively) and fixed on a stereotaxic frame. In 2 µL of native EMEM (ATCC), 4 × 10^4^ cells of U-87MG (ATCC) were injected into the right frontal lobe using an infusion syringe pump (Harvard Apparatus, Holliston, MA, USA) mounted with a Hamilton syringe (26S gauge needle). The injection coordinates were 2.1 mm lateral and 0.5 mm posterior from the bregma, and 2.6 mm deep from the outer border of the cranium [12]. The tumor size monitoring was performed via MRI (see Section 2.3).

### 2.2. Mannitol-Induced Hyperosmotic BBB Disruption

When the tumor size reached 7 ± 1 mm^3^, osmotic shock was induced with an intracarotid infusion of a hypertonic solution of mannitol 25% v/m (the control group received NaCl 0.9%). Mice were anesthetized with isoflurane mixed with air (2.5% for induction, 1.5% for maintenance). The common carotid artery (CCA) bifurcation was isolated. A polyethylene microcatheter (PE 10, inner diameter 0.28 mm and outer diameter 0.61 mm, BD Intramedic^TM^, Sparks, NV, USA) was inserted into the CCA via a small arteriotomy and moved into the internal carotid artery for an infusion of warm and filtered mannitol 25% or normal saline (200 µL/min for 1 min) [13]. 

### 2.3. MRI

MRI was performed using a 11.7 T Bruker Biospec MRI system (Bruker, Ettlingen, Germany) equipped with a ^1^H quadrature transmit/receive birdcage coil (21 mm inner diameter, RAPID Biomedical, Rimpar, Germany). Mice were anesthetized with isoflurane mixed with air (2.5% for induction, 1.5% for maintenance). Animals were covered with a heating blanket and their temperature was monitored. A pressure pad was used to monitor the respiration rate.

Anatomical images were obtained using T_2_-weighted rapid acquisition with a refocused echo (RARE) sequence (echo time = 30 ms; repetition time = 2500 ms; number of slices = 25; field of view = 20 mm × 20 mm; matrix size = 200 × 200; resolution = 0.1 mm × 0.1 mm; slice thickness = 0.3 mm; acquisition time = 5 min 20 s; averages = 8). Tumor volume was determined from a manually drawn region of interest (ROI) using Paravision 6.0.1 software (Bruker BioSpin) on day 14 following tumor induction, and then daily until the tumor size reached 7 ± 1 mm^3^.

For DCE-MRI acquisition, T_1_-weighted gradient echo images were obtained via a fast low-angle shot (FLASH) sequence (echo time = 1.4 ms; repetition time = 11.719 ms; flip angle = 10.0°; field of view = 20 mm × 20 mm; matrix size = 128 × 128; resolution = 0.156 mm × 0.156 mm; slice thickness = 0.9 mm; averages = 1; total acquisition time = 22 min 40 s). A set of 450 scans with a temporal resolution of 3.02 s was acquired, with Gadolinium-DOTA (Dotarem^®^ 0.5 mol/mL; Guerbet, Villepinte, France) administered intravenously at a dose of 0.29 mmol/kg after the 10th scan over 5 s. DCE-MRI acquisition was performed 24 h before (day 0), 5 min after (day 1), and 24 h (day 2) after the injection of mannitol 25% or saline.

### 2.4. Histological Study

Evans blue dye (EB: 2% in normal saline; Alfa Aesar, Haverhill, MA, USA) was intravenously injected (3 mL/kg) 5 min after Mannitol or NaCl intra-carotid injection. Thirty minutes later, the mice were euthanized and intracardially perfused with paraformaldehyde (PFA) 4% to discard all the remaining dye in the blood vessels and fix the tissue. Brains were then removed, fixed overnight in PFA 4%, cryoprotected in sucrose 20%, included in optimal cutting temperature compound (OCT, Sakura Finetek, Alphen aan den Rijn, The Netherlands), and kept at −80 °C. Cryostat 30 µm sections were counterstained with diamidino-2-phenylindole (DAPI, Thermofisher, Waltham, MA, USA) and examined under a fluorescence microscope slide scanner (Panoramic 250 Flash III, 3DHistech, Budapest, Hungary) with DAPI and Cyanine 5 (Cy5) filter [14,15]. 

### 2.5. Image Processing and Statistical Analyses

DCE-MRI data were analyzed using in-house software written in Matlab (version 9.6). Regions of interest (ROIs) were manually delineated. We considered ROI T_1_ as the delineation of the entire tumor area using T_1_-weighted images with Gd-DOTA as the contrast agent, ROI T_2_ as the delineation of the tumor bulk area using T_2_-weighted anatomical images, and ROI Delta as the ROI T_1_ from which we subtracted ROI T_2_ in order to cover the margins of the tumor. In this way, we were able to study the hemodynamic parameters for the whole tumor region but also for the margins of the tumor.

The hemodynamic parameters were computed using a two-compartmental model, the extended Tofts model [16]. This model takes into account the contribution of the vascular compartment, which is not negligible in tumors.

(1) $$C_{t}\left(t\right)=v_{p}\times C_{p}\left(t\right)+K^{trans}\int_{0}^{t}C_{p}\left(\tau \right)e^{-k_{ep}(t-\tau )}d\tau$$

where K^trans^ is the volume transfer constant between blood plasma and extravascular extracellular space (EES) [min^−1^], v_p_ is the blood plasma volume per unit volume of tissue, and k_ep_ is the flux rate constant between EES and blood plasma [min^−1^] [17]. V_e_ is the EES volume per unit volume of tissue, calculated as follows:

(2) $$v_{e}=K^{trans}/k_{ep}$$

We were also interested in AUC60 and AUC90 corresponding to the area under the curve (AUC) of contrast agent concentration as a function of time from 0 to 60 or to 90 s.

Histological images were analyzed using Qupath (version 0.3.2) [18]. The tumor ROI and the Evans blue diffusion ROI were manually delineated. 

For statistical analyses, two-way ANOVA tests (Tukey’s test) and t-tests were performed using GraphPad Prism (version 9.1.2), with *p*-values < 0.05 (*), *p* < 0.01 (**), *p* < 0.001 (***), and *p* < 0.0001 (****) considered as the levels of significance. The results are presented as mean ± standard deviation (SD).

## 3. Results

### 3.1. Histological Study

**Figure 2:**
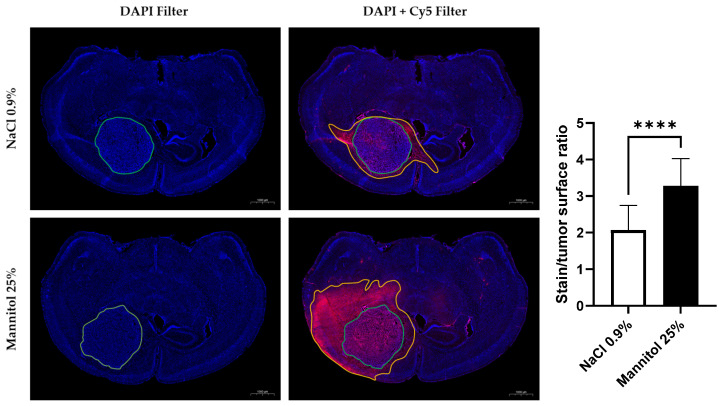
Assessment of diffusion of contrast agent outside of the tumor area via histology with Evans blue dye. Representative histological images of brain sections from a control mouse (treated with NaCl 0.9%, top row) and a mouse treated with osmotic shock (mannitol 25%, bottom row). The tumor area is encircled in green and the area of diffusion of EB is delineated in orange. The right panel shows the ratio of the surface stained with EB on the tumor surface for mice treated with osmotic shock (*n* = 4) and control mice (*n* = 4). The fluorescent dye diffused more largely outside of the tumor area after osmotic shock than in untreated tumors. The results are expressed as means ± SD. **** *p* < 0.0001.

BBB integrity was assessed by perfusing mice with EB dye, a fluorescent vascular leakage marker. On the brain sections treated with osmotic shock, the fluorescent dye diffused more largely outside of the tumor area than on the brain sections of the untreated tumors (Figure 2). We observed that the surface stained with EB was about two times larger than the tumor surface area (1.93 ± 0.43, mean ± SD, *n* = 4) in the control mice (treated with saline). The EB stained/tumor surface ratio in the group of mice receiving osmotic shock (3.282 ± 0.74; mean ± SD, *n* = 4) was significantly higher (*p* < 0.0001) than in the control group receiving saline, a result consistent with an opening of the BBB.

### 3.2. MRI Studies

#### 3.2.1. T_1_/T_2_ Tumor Surface Ratio

**Figure 3:**
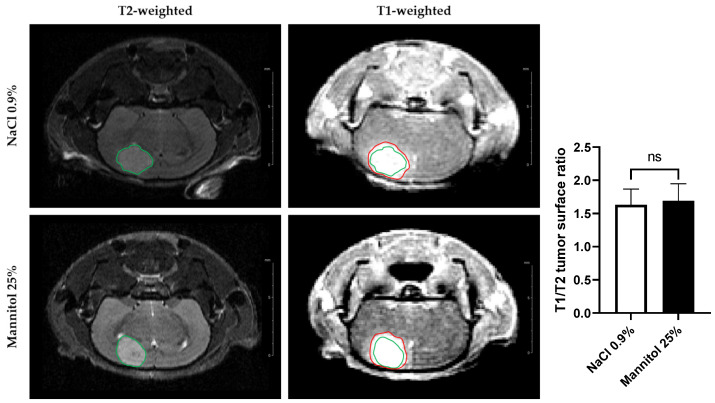
Assessment of diffusion of contrast agent outside of the tumor area using MRI. MRI images of brains from control mice (receiving NaCl 0.9%, top row) and mice treated with osmotic shock (receiving mannitol 25%, bottom row), with T_2_-weighted contrast (**left**) and T_1_-weighted contrast after administration of Gd-DOTA (**right**). The T_2_-weighted images allow the anatomical delineation of the tumor (encircled in green) and the post-contrast T_1_-weighted images allow the assessment of vascular leakage (encircled in red). The right panel shows the ratio between the T_1_ and T_2_ surface areas. Using this procedure, no significant difference in BBB permeability was observed between tumors receiving saline (*n* = 5) or mannitol (*n* = 7). The results are expressed as means ± SD.

BBB permeability was additionally evaluated using MRI. Here, we determined the anatomical tumor size using T_2_-weighted contrast images and diffusion of the tracer inside and outside of the tumor using T_1_-weighted images after the administration of Gd-DOTA used as a contrast agent (Figure 3). There was no significant difference (*p* > 0.05) between the T_1_/T_2_ surfaces ratio of the treated (1.68 ± 0.24, mean ± SD, *n* = 7) and untreated group (1.63 ± 0.21, mean ± SD, *n* = 5). These results indicate that this MRI assessment was unable to detect any difference in the BBB permeability between these two groups.

#### 3.2.2. DCE-MRI

To further investigate BBB disruption, we also performed DCE-MRI to provide hemodynamic parameters such as contrast agent efflux transfer constant (K^trans^), contrast agent reflux transfer constant (k_ep_), the intravascular volume fraction (v_p_), the extravascular volume fraction (v_e_), and the area under the curve of contrast agent concentration as a function of time from 0 to 60 or to 90 s (AUC60 and AUC90). These parameters were analyzed in two different regions of interest (ROIs) of the tumor: the ROI T_1_ corresponding to the whole tumor region, and the ROI Delta corresponding to the margin tumor area.

**Figure 4:**
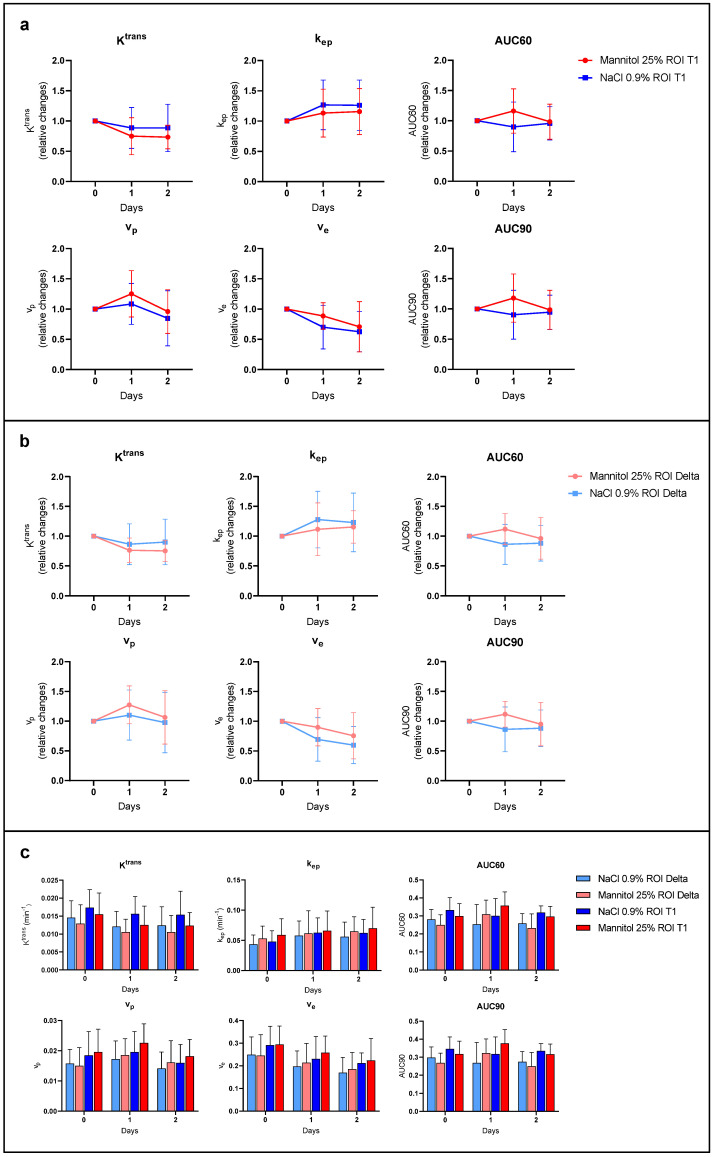
Hemodynamic parameters measured via DCE-MRI study. (**a**) Relative changes in tumor hemodynamic parameters (K^trans^, k_ep_, v_p_, v_e_, AUC60, and AUC90) between mice treated with osmotic shock (dark red) and control mice (dark blue) using ROI T_1_; (**b**) relative changes in tumor hemodynamic parameters between mice treated with osmotic shock (light red) and control mice (light blue) using ROI Delta corresponding to the margin tumor area; note that the changes observed in the margins (**b**) are rather comparable to the changes observed in the core of the tumor (**a**), with the variability being generally larger in the tumor margins; (**c**) tumor hemodynamic parameter values between mice treated with osmotic shock using ROI Delta (light red) and using ROI T_1_ (dark red), and control mice using ROI Delta (light blue) and ROI T_1_ (dark blue). DCE-MRI acquisition was performed 24 h before (day 0), 5 min after (day 1), and 24 h (day 2) after the injection of mannitol 25% or saline. There was no significant difference in the tumor hemodynamic parameters between tumors receiving mannitol (*n* = 7) and saline (*n* = 5), whatever the studied parameter and ROI. The results are expressed as means ± SD.

**Figure 5:**
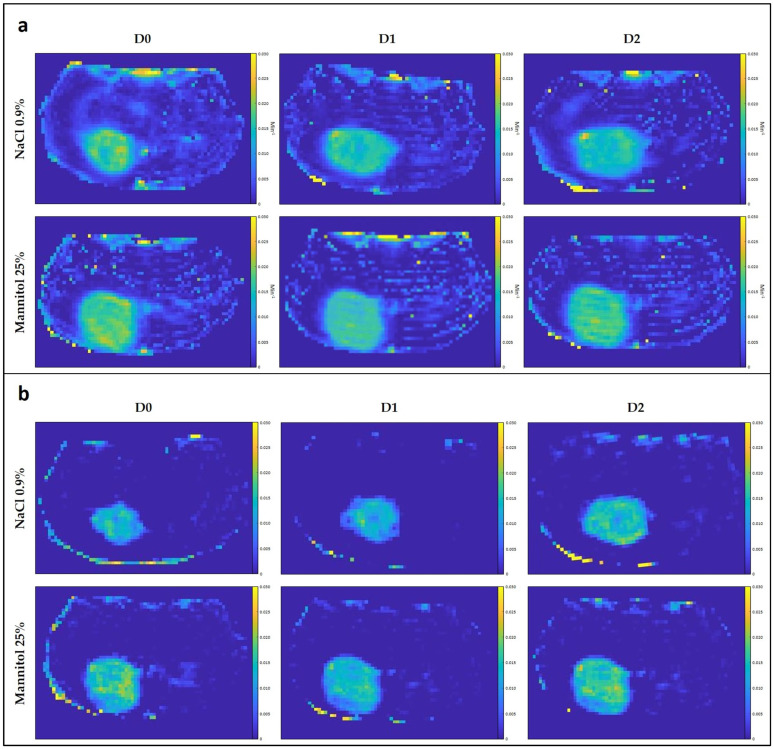
DCE-MRI parametric maps. Illustration of typical K^trans^ (**a**) and v_p_ (**b**) maps obtained from an untreated (NaCl 0.9%) and a treated mouse (mannitol 25%) each with Gd-DOTA. DCE-MRI acquisition was performed 24 h before (day 0), 5 min after (day 1), and 24 h (day 2) after the injection of mannitol 25% or saline.

We did not observe any significant difference in the tumor hemodynamic parameters between tumors in mice treated with mannitol (*n* = 7) or saline (*n* = 5), whatever the studied parameter and the studied ROI (Figure 4 and Figure 5).

## 4. Discussion

While the BBB is generally disrupted at the site of the tumor bulk in glioblastoma, the BBB remains intact in the infiltrating part of the tumor margins, that is, at the origin of subsequent tumor recurrence [19,20,21,22]. This feature limits the conventional systemic delivery of many chemotherapy drugs and allows residual tumor cells to escape to cytotoxic treatments [23]. Several strategies have emerged to overcome this limited accessibility of drugs to residual tumor cells by temporarily and reversibly opening the BBB. Among the strategies, our present study has been focused on the use of the intraarterial infusion of a hypertonic solution. In preclinical studies, EB staining is often used to assess BBB permeability/integrity, including in glioblastoma models [24,25,26]. While this histological tool is highly effective in preclinical models, it requires the sacrifice of the animal and is obviously useless for clinical applications. To monitor treatment-induced changes in BBB permeability, DCE-MRI is particularly attractive as contrast-enhanced MRI is systematically used for the characterization of brain tumors in patients. Previous studies have shown that contrast-enhanced MRI was useful in preclinical models to assess changes in BBB permeability and to evaluate the effect of strategies opening the BBB [13,27,28]. Of note, most studies focused on the BBB opening in the brain parenchyma. A few examples evaluated ultrasound-mediated BBB opening in glioblastomas [29,30,31], but, as far as we know, none evaluated the ability of DCE-MRI to monitor osmotic-shock-induced BBB opening in brain tumors. In our study, particular attention was paid to the ability of the treatment to enlarge the delivery of compounds into the margins of the tumor. For this purpose, we compared areas accessible to BBB-impermeable agents with the anatomical areas of the tumors, with or without osmotic treatment. 

In the histological studies, we observed that the area accessible to EB was 93% larger than the anatomic area in the group of mice receiving saline (control mice) (Figure 2). This suggests that the BBB is already permeable in regions surrounding the bulk tumor in the U-87MG mouse glioblastoma model. The diffusion of EB significantly increased after the infusion of 20% mannitol solution in the carotid artery: the accessible area to the dye was 3.28 times the area covered by the bulk tumor, as defined via DAPI staining (Figure 2). This increased diffusion capability could be particularly interesting to exploit in order to increase the delivery of anticancer drugs, whatever their mode of action. 

To mimic the endpoints measured via histology in the MRI study, we compared T_2_-weighted images (corresponding to the anatomical image of the bulk tumor) and T_1_-weighted images after Gd-DOTA administration (corresponding to areas accessible by the BBB-impermeable contrast agent). In the group of mice receiving saline (control mice), the area accessible to Gd-DOTA visualized in the T_1_-weighted images was 68% larger than the bulk area of the tumor seen in the T_2_-weighted images (Figure 3). The area accessible to Gd-DOTA was not significantly altered for the group receiving the mannitol infusion (Figure 3). In contrast to the EB assay, non-invasive MRI using Gd-DOTA thus seemed unable to tackle these changes in BBB permeability in the surrounding margins of the tumor. In addition to the focus on diffusion patterns outside of the tumor bulk area, we sought to identify possible changes in intratumoral and marginal hemodynamics. For this purpose, we interrogated possible changes in K^trans^, k_ep_, v_e_, v_p_, AUC60, and AUC90 in these tumor regions. There were no significant relative changes in these parameters compared to the basal values recorded one day before the treatment (Figure 4).

The differences in the results obtained through both methods regarding the assessment of BBB opening deserve discussion. It is first important to note that the values recorded via histology or MRI cannot be superimposed. First is the invasive nature of histology in terms of repeating the measurement on the same animal: the histological images obtained in the control and treated mice came from different cohorts of animals, while the MR images were measured longitudinally on the same animals. More importantly, it is important to realize that tracers differ in their molecular and biodistribution properties. Fluorescent EB (960 Da, molecular weight) strongly binds to albumin (68 kDa), while Gd-DOTA (580 Da) is a highly hydrophilic compound that does not bind to albumin. These properties mean that they have differences in their ability to cross vessel fenestrations. Gd-DOTA can diffuse easily in the extracellular compartment of normal vessels, except for those located in the BBB due to inter-endothelial tight junctions. In contrast, due to its binding to albumin, EB remains in the vessels, is unable to cross small fenestrations in the vascular wall, and can only cross large fenestrations. As osmotic shock induces a change in tight junctions between endothelial cells, we can assume that the observed change in permeability would be more pronounced for larger molecules (complex EB-albumin) than for smaller molecules (Gd-DOTA) that were already able to cross small fenestrations of the damaged tumoral BBB. Comparative studies have indeed reported differences in glioblastoma accumulation for small Gd complexes and radiolabeled albumin measured via PET [32]. In the future, it could be interesting to test the size-dependent ability to report on BBB opening using larger molecular entities or gadolinium-based contrast agents with high affinity for albumin, such as gadobenate (Gd-BOPTA), that would better mimic the distribution behavior of EB [33]. Moreover, it is important to notice that both methods present differences in terms of sensitivity and resolution. Indeed, the optical fluorescent method has a higher sensitivity of detection of probes informing on BBB permeability and much higher spatial resolution than the MRI method.

Regarding the DCE-MRI study, it is likely that the changes in hemodynamic parameters could be too subtle to be detected through the use of the pharmacokinetic model (the extended Tofts model). This model is a two-compartmental model widely used to study hemodynamic parameters in tumors [17,34]. It takes into account the contribution of the vascular compartment which is not negligible in tumors. Several studies have used this model to describe variations in hemodynamic parameters (K^trans^, k_ep_, v_e_, and v_p_) after the disruption of the BBB [35,36,37,38,39]. Here, however, we were unable to tackle any variation in these parameters using the extended Tofts model. Measuring subtle BBB leakage using DCE-MRI presents unique challenges [40,41,42]. Among the kinetic models described in the literature, the Patlak model has been reported to detect subtle changes in hemodynamics associated with BBB disruption [40,41]. This model seems to be appropriate for studying low-level blood-brain barrier leakage where the back-flux from the interstitium to the capillaries is negligible [42,43,44,45]. In the tumor core of the GB, the BBB is damaged and the extended Tofts model seems to be more appropriate as the back-flux is not negligible. However, in further studies, it could be interesting to apply the Patlak model in the margins of the tumor where the BBB is less fenestrated and where the back-flux from the interstitium can be neglected. The parameters AUC60 and AUC90 that are model-independent could have potentially hinted a change in contrast agent uptake. While there was a trend for an increased AUC60 and AUC90, the changes were not significant.

It is important to highlight that the anesthetic regimen used in the present study may have played a role in our assessment of hemodynamics. We used isoflurane because previous studies have shown that this mode of anesthesia preserved the oxygenation in most tissues and peripheric tumors [46,47]. However, in the brain, several reports have suggested that isoflurane presents a vasodilatory effect that can increase baseline cerebral blood flow. This effect of isoflurane may decrease the vasodilatory reserve that can be recruited, as reported in functional MRI studies [48,49,50,51]. Therefore, isoflurane could have potentially affected our ability to see hemodynamic changes at the brain tumor level. However, we should notice that isoflurane was used for both EB and MRI assessments. As we observed, regarding the permeabilization of the BBB using EB, it is unlikely that our inability to see this phenomenon using MRI was due to the sole effect of isoflurane.

## Data Availability

All data will be provided upon e-mail request.
